# Supplementary material for: High-speed motility originates from cooperatively pushing and pulling flagella bundles in bilophotrichous bacteria
Source: eLife. 2020 Jan 28;9:e47551. doi: 10.7554/eLife.47551 (PMC7010408; doi:10.7554/eLife.47551)
Supplement: Transparent reporting form [file elife-47551-transrepform.docx]

***eLife’s* transparent reporting form**

We encourage authors to provide detailed information *within their submission* to facilitate the interpretation and replication of experiments. Authors can upload supporting documentation to indicate the use of appropriate reporting guidelines for health-related research (see [EQUATOR Network](http://www.equator-network.org/%20)), life science research (see the [BioSharing Information Resource](https://biosharing.org/)), or the [ARRIVE guidelines](http://www.plosbiology.org/article/info:doi/10.1371/journal.pbio.1000412) for reporting work involving animal research. Where applicable, authors should refer to any relevant reporting standards documents in this form.

If you have any questions, please consult our Journal Policies and/or contact us: [editorial@elifesciences.org](mailto:editorial@elifesciences.org).

**Sample-size estimation**

- You should state whether an appropriate sample size was computed when the study was being designed
- You should state the statistical method of sample size computation and any required assumptions
- If no explicit power analysis was used, you should describe how you decided what sample (replicate) size (number) to use

Please outline where this information can be found within the submission (e.g., sections or figure legends), or explain why this information doesn’t apply to your submission:

No explicit power analysis was used as only helix parameters and reorientation events were measured.

**Replicates**

- You should report how often each experiment was performed
- You should include a definition of biological versus technical replication
- The data obtained should be provided and sufficient information should be provided to indicate the number of independent biological and/or technical replicates
- If you encountered any outliers, you should describe how these were handled
- Criteria for exclusion/inclusion of data should be clearly stated
- High-throughput sequence data should be uploaded before submission, with a private link for reviewers provided (these are available from both GEO and ArrayExpress)

Please outline where this information can be found within the submission (e.g., sections or figure legends), or explain why this information doesn’t apply to your submission:

(RESULTS:) A new cell culture was harvested for each of the six different experiments (100 fps tracking for reorientation event detection, 400 fps tracking for helix parameter determination, 1640 fps dark-field for cell tracking, dark-field flagella imaging).

**Statistical reporting**

- Statistical analysis methods should be described and justified
- Raw data should be presented in figures whenever informative to do so (typically when N per group is less than 10)
- For each experiment, you should identify the statistical tests used, exact values of N, definitions of center, methods of multiple test correction, and dispersion and precision measures (e.g., mean, median, SD, SEM, confidence intervals; and, for the major substantive results, a measure of effect size (e.g., Pearson's r, Cohen's d)
- Report exact p-values wherever possible alongside the summary statistics and 95% confidence intervals. These should be reported for all key questions and not only when the p-value is less than 0.05.

Please outline where this information can be found within the submission (e.g., sections or figure legends), or explain why this information doesn’t apply to your submission:

The helix parameter determination was performed automatically on 400 fps tracks extending a duration of 0.4 s, with a mean-square-displacement of at least 10 µm^2^ and a mean opening angle between all consecutive velocity vectors of less than 60 ° to exclude strongly irregular tracks. The same exclusion parameters have been used for the reorientation angle analysis in the 100 fps tracks. (MATERIALS AND METHODS: CELL AND FLAGELLA TRACKING SOFTWARE)

The 400 fps data with exclusion criteria resulted in 65 tracks (RESULTS SECTION) that were used for helix parameter measurements. 4237 events (FIGURE 4 LEGEND, SI FIGURE SI3) could be detected using the 100 fps data and an automated swimming track analysis script, that was tested against simulated data. The algorithm for 3D track event detection was validated (SI FIGURE S2) using Langevin-simulations of single active Brownian particles with defined mean reorientation angles, velocities, mean run times and mean run lengths.

(For large datasets, or papers with a very large number of statistical tests, you may upload a single table file with tests, Ns, etc., with reference to sections in the manuscript.)

**Group allocation**

- Indicate how samples were allocated into experimental groups (in the case of clinical studies, please specify allocation to treatment method); if randomization was used, please also state if restricted randomization was applied
- Indicate if masking was used during group allocation, data collection and/or data analysis

Please outline where this information can be found within the submission (e.g., sections or figure legends), or explain why this information doesn’t apply to your submission:

Does not apply, since no experimental groups were differentiated.

**Additional data files (“source data”)**

- We encourage you to upload relevant additional data files, such as numerical data that are represented as a graph in a figure, or as a summary table
- Where provided, these should be in the most useful format, and they can be uploaded as “Source data” files linked to a main figure or table
- Include model definition files including the full list of parameters used
- Include code used for data analysis (e.g., R, MatLab)
- Avoid stating that data files are “available upon request”

Please indicate the figures or tables for which source data files have been provided:

3D track files have been uploaded to datadryad.org together with relevant MatLab scripts.
